# A multi-site pilot randomized clinical trial of the Treatment and Education Approach for Childhood-onset Lupus (TEACH) program: study design and COVID-19 adaptations

**DOI:** 10.1186/s12969-023-00835-6

**Published:** 2023-06-23

**Authors:** Natoshia R. Cunningham, Alaina Miller, Samantha L. Ely, Mallet R. Reid, Ashley Danguecan, Sarah I. Mossad, Luana Flores Pereira, Khalid Abulaban, Elizabeth Kessler, Natalie Rosenwasser, Kabita Nanda, Tamar Rubinstein, Mathew Reeves, Sara Ahola Kohut, Jennifer Stinson, Tala El Tal, Deborah M. Levy, Linda Hiraki, Emily A. Smitherman, Andrea M. Knight

**Affiliations:** 1 Department of Family Medicine, Michigan State University, 15 Michigan St NE, Grand Rapids, MI 49503 US; 2 School of Professional Psychology, Wright State University, 3640 Colonel Glenn Hwy, Dayton, OH 45435 US; 3 Present address: Department of Psychiatry and Behavioral Neuroscience, Wayne State University School of Medicine, 3901 Chrysler Service Drive, Detroit, MI 48201 US; 4 Department of Psychology, Michigan State University, 316 Physics Rd, East Lansing, MI 48824 US; 5 Department of Psychology, The Hospital for Sick Children, 555 University Ave, Toronto, ON M5G 1X8 Canada; 6 Division of Rheumatology, The Hospital for Sick Children, 555 University Ave, Toronto, ON M5G 1X8 Canada; 7 The Hospital for Sick Children, Mental Health and Neurosciences Program, 555 University Ave, Toronto, ON M5G 1X8 Canada; 8 Pediatric Rheumatology, Helen DeVos Children’s Hospital, 35 Michigan St NE, Grand Rapids, MI 49503 US; 9 Rheumatology, Seattle Children’s Hospital, 4800 Sand Point Way NE, Seattle, WA 98105 US; 10 Division of Pediatric Rheumatology, Department of Pediatrics, Children’s Hospital at Montefiore/Albert Einstein College of Medicine, 3415 Bainbridge Ave, NY 10467 Bronx, US; 11 Michigan State University, Department of Epidemiology and Biostatistics, 909 Wilson Rd, MI 48824 East Lansing, US; 12 Child Health Evaluative Sciences, The Hospital for Sick Children, 555 University Ave, ON M5G 1X8 Toronto, Canada; 13 University of Alabama at Birmingham, The Children’s Hospital CPP N G10, 1600 7 Ave S Birmingham, Birmingham, AL 35223-1711 USA

**Keywords:** Childhood-onset systemic lupus erythematosus (cSLE), RCT- randomized clinical trial, Fatigue, Depressive symptoms, Pain, Cognitive behavioral therapy

## Abstract

**Background:**

Childhood-onset Systemic Lupus Erythematosus (cSLE) is an autoimmune disease associated with fatigue, mood symptoms, and pain. Fortunately, these symptoms are potentially modifiable with psychological intervention such as cognitive-behavioral therapy (CBT). The Treatment and Education Approach for Childhood-onset Lupus (TEACH) program is a CBT intervention developed to target these symptoms for adolescents and young adults with cSLE. This pilot randomized controlled trial (RCT) aims to determine the feasibility and effect of TEACH for youth with cSLE. Adjustments to the study protocol following the COVID-19 pandemic are also described.

**Methods:**

This two-arm multisite RCT will explore the feasibility (primary outcome) and effect (secondary outcome) of a remotely delivered TEACH protocol. Participants will be randomized to a six-week remotely delivered TEACH program plus medical treatment as usual (TAU) or TAU alone. We will include patients ages 12–22 years presenting to rheumatology clinics from six sites. Validated measures of fatigue, depressive symptoms, and pain will be obtained at baseline and approximately eight and 20 weeks later. Protocol adjustments were also made due to the COVID-19 pandemic, in collaboration with the investigative team, which included patients and caregivers.

**Conclusions:**

Findings from this multi-site RCT aim to document the feasibility of TEACH and provide an estimate of effect of a remotely delivered TEACH protocol on fatigue, depression, and pain symptoms in youth with cSLE as compared to standard medical treatment alone. This findings may positively impact clinical care for patients with cSLE. Clinical trials.gov registration: NCT04335643.

## Introduction

Childhood-onset Systemic Lupus Erythematosus (cSLE) is a chronic, multisystem, autoimmune disease disproportionately impacting females of color [1]. Individuals with cSLE often experience fatigue, depressive symptoms, and pain, which negatively impact health-related quality of life [2, 3]*.* One-third of youth with cSLE have a psychological disorder (e.g., depression or anxiety), which is higher than rates observed in the general population [4, 5]. Additionally, anxiety and depression in children with chronic illnesses increased during the COVID-19 pandemic [6].

Cognitive behavioral therapy (CBT) is the gold standard for treatment of fatigue [7], depression [8, 9], and pain [10, 11] in youth [12] and has improved mental health functioning in adults with SLE [13]. Further, CBT is beneficial for children with diabetes [14], juvenile fibromyalgia [15], and inflammatory bowel diseases [16], with improvements in mood symptoms, adjustment, and medication adherence [17]. Therefore, CBT [8–11]) may improve outcomes for youth with cSLE [18].

Our research team developed the Treatment and Education Approach for Childhood-Onset Lupus (TEACH), a tailored CBT for youth with cSLE targeting mood symptoms, fatigue, and pain [18]. TEACH was originally designed as a six-session, in-person individual treatment. Pilot testing at a single site showed TEACH was potentially beneficial; however, only 50% of patients agreed to participate [18]. Furthermore, barriers (e.g., travel to appointments, patient burden) to treatment were reported. This signaled a critical need to partner with patients and families to refine the intervention to increase access to care.

An advisory co-investigative team including five cSLE patients and caregivers collaborated to refine TEACH to be delivered remotely. The team then received funding from the Childhood Arthritis and Rheumatology Research Alliance – Arthritis Foundation (CARRA-AF) to conduct a pilot randomized clinical trial (RCT) to investigate the feasibility of the *remotely delivered* TEACH plus standard medical treatment as usual (TAU) and its effect on fatigue, depressive symptoms, and pain as compared to TAU alone. It is hypothesized that TEACH + TAU will be feasible and more effective at reducing these symptoms compared to TAU alone. This paper details the study protocol and modifications made as a result of the COVID-19 pandemic.

### Study aims and hypotheses

#### Primary outcomes

To determine the feasibility of a remotely-delivered TEACH program for youth with cSLE. Program feasibility will be based on recruitment/retention rates and qualitative participant interviews. It is hypothesized that > 65% of people approached will agree to participate (H1) and > 80% will complete the TEACH protocol (H2). Additionally, it is hypothesized that TEACH participants will support program feasibility through qualitative interviews (H3).

#### Secondary outcomes

To examine the effect of TEACH in youth with cSLE. It is hypothesized that fatigue (H1), depressive symptoms (H2), and pain (H3) will significantly decrease for the TEACH + TAU group at post assessment compared to the TAU group.

#### Exploratory outcomes

Exploratory outcomes include health-related (e.g., disease activity/severity, health-related quality of life, pain interference, medication adherence) and mental health-related (e.g., anxiety, resilience; ACEs; COVID-related participant distress; and caregiver mental health) outcomes will be collected, in addition to daily diaries of fatigue, mood symptoms and pain. Long term outcomes (20 weeks from baseline) will also be explored. The impact of sociodemographic factors on treatment response will also be investigated.

## Methods

### Study design

**Fig. 1:**
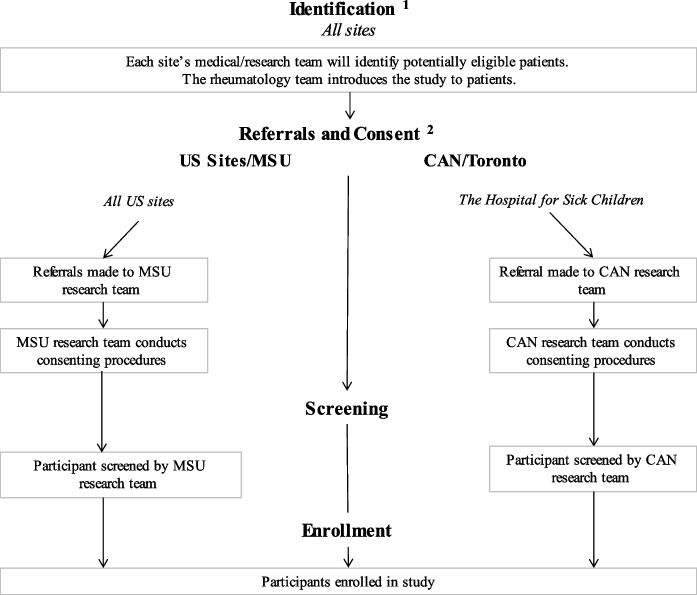
Referral, screening, and enrollment by referral site. Each rheumatology sites’ medical/research team will identify potentially eligible participants. US patients will be referred to research staff at MSU for consenting (if needed), screening, and enrollment. CAN patients will complete these study activities (e.g., consenting, screening, enrollment) with research staff at their home site, Toronto Hospital for Sick Children

This two-arm, international multi-site RCT will examine the feasibility and effect of a remotely delivered TEACH protocol in reducing symptoms in youth with cSLE. Six children’s hospitals (five in the United States [US], one in Canada [CAN]) plan to recruit participants. The anticipated participating sites expanded to this total number following the onset of the COVID-19 pandemic. All sites have established pediatric rheumatology programs with a substantial referral base. Two primary locations in the US (Michigan State University [MSU], led by NRC) and CAN (The Hospital for Sick Children, led by AMK) will coordinate study activities. Each primary location houses site PIs, interventionists, and research coordinators. MSU will receive referrals from the US-based sites: Helen DeVos Children’s Hospital, Seattle Children’s Hospital), Montefiore Medical Center/The Children’s Hospital at Montefiore), University of Alabama-Birmingham/Children’s of Alabama), and Cincinnati Children’s Hospital and Medical Center (see Fig. 1).

Each referring site is anticipated to enroll between 5 and 20 participants, with an anticipated total of 75 participants. Across all sites, we expect a retention rate of 80% and a final sample size of *n* = 60 participants completing the post assessment. A longitudinal design will be employed with assessments at baseline, post-assessment (eight weeks later), and 20-week follow-up from baseline. Feasibility (primary outcome), symptom response (secondary outcome), and additional factors will be assessed at each time point (see below).

### Eligibility criteria

Seventy-five participants ages 12–22 years diagnosed with cSLE from participating rheumatology clinics will be enrolled.

*Inclusion criteria* will include patients who must: be diagnosed with cSLE, meeting the revised American College of Rheumatology Classification Criteria for SLE by age 18 [19]; be between the ages of 12 and 22 years; have clinical elevations in fatigue or depressive symptoms, or pain (see Measures section); have English language proficiency; and those under age 18 must be accompanied by a primary caregiver with English language proficiency willing to participate.

*Exclusion criteria* include patients with: other chronic medical conditions (e.g., juvenile idiopathic arthritis); a documented developmental delay, severe cognitive impairment, thought disorder; or an untreated major psychiatric illness (e.g., bipolar disorder, psychosis, severe depression, or active suicidal ideation (see Measures section)). In cases where patient safety is a concern, study staff will be notified immediately and a risk assessment will be conducted with oversight from a study psychologist. Parents will be informed for youth under 18 years, and appropriate safety measures (e.g., outpatient referrals, hospitalization) will be taken. Of note, the depressive symptom exclusionary criterion was adjusted by the study team given increased depressive symptoms in the cSLE population following the onset of COVID-19 [6]. Specifically, cut-off scores on the depression inventory were adjusted from T-score > 80 to T-score > 90 in August of 2021.

### Study procedures

**Fig. 2:**
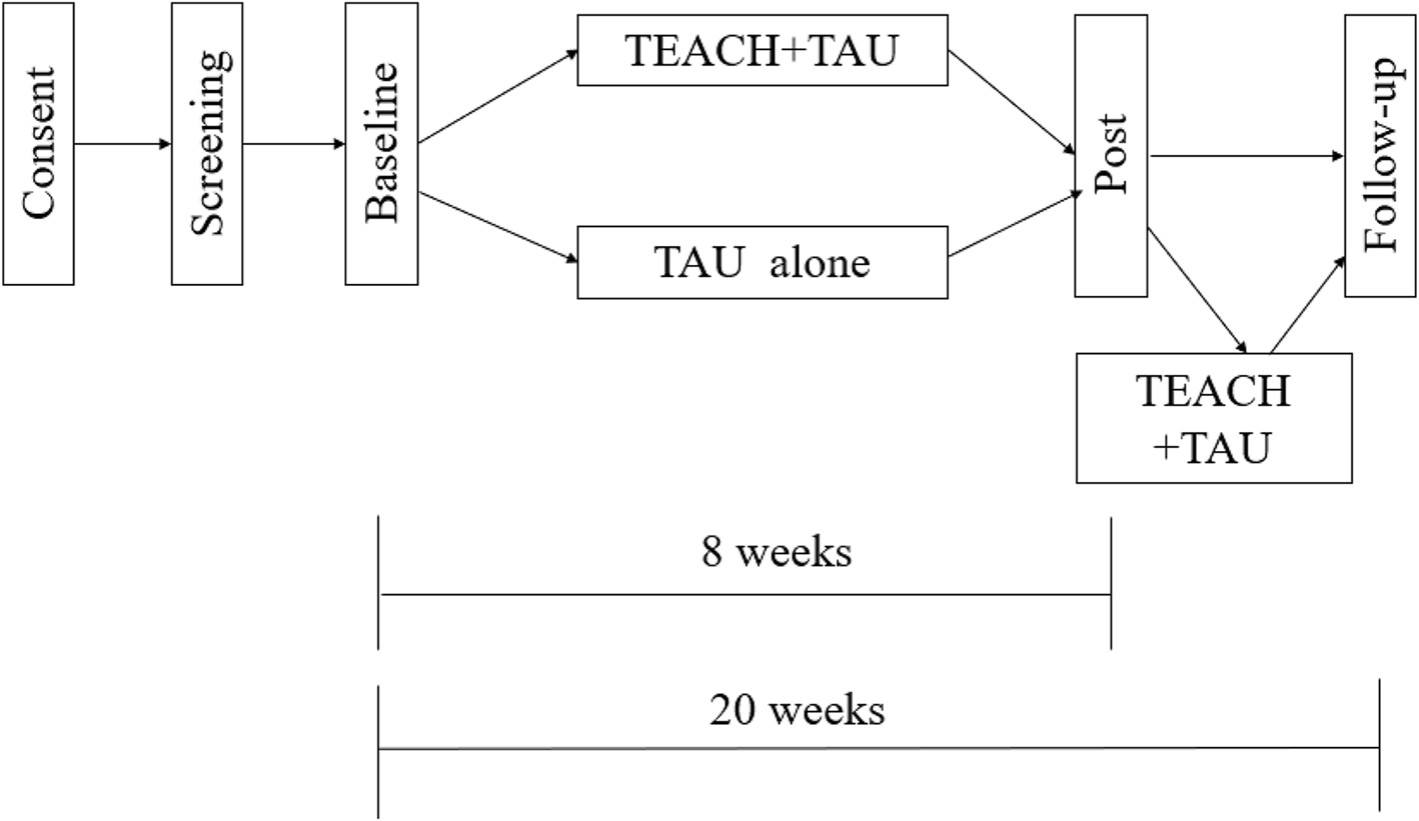
Study Design and Timeline. After consenting, participants will be screened for study eligibility. If eligible, participants will complete a baseline assessment and will then be randomized to TEACH + TAU or medical TAU alone. All participants will complete a post assessment approximately eight weeks after baseline. If they were randomized to TAU alone, they will be able to participate in TEACH + TAU after the post assessment is completed. An additional follow-up (20 weeks from baseline) will also be conducted with all participants

Physicians will identify youth who meet criteria for cSLE, introduce the study, and then study staff will speak with the family to obtain consent/assent. If study staff are not physically present, virtual consent procedures will be conducted via phone and REDCap’s eConsent Framework to minimize patient risk. After consent, study staff will conduct a psychosocial screening to determine eligibility (~ 5 min), as well as a baseline assessment with qualifying youth (see Fig. 1). Eligible patients will then be randomized to receive TEACH + TAU or TAU alone (See Fig. 2). Approximately eight and 20 weeks later, all subjects will be re-assessed on study outcomes. If significant symptom levels remain (or if family requests additional care), participants will be offered appropriate referrals. Following the post assessment, those randomized to TAU will then be eligible to receive TEACH+TAU (see Fig. 2). Subjects will receive up to $100 in Amazon gift cards ($50 at baseline and $50 at the eight-week assessment; no compensation is offered for 20-week assessment). Participants at the CAN site will receive research volunteer hours at the 20-week visit.

### Randomization and blinding

Eligible participants will be randomized to receive either TEACH + TAU or TAU alone in this open trial. Randomization scheduling will be computer-generated by a biostatistician at MSU and stratified based on the two main study sites and two age groups (< 18, 18 +), with a 1:1 ratio for each condition. We will also use blocking to keep the ratio of TEACH + TAU vs. TAU approximately equal throughout the trial. The study team including the site recruiters will be masked to the block size(s). Post assessment and follow up measures will be collected autonomously using online methods, allowing for blinded collection of outcome data irrespective of group assignment.

### Assessments & outcome measures

Study assessments will occur at baseline, eight weeks, and 20-weeks following the baseline assessment. Psychometrically validated instruments (self-report unless otherwise indicated) sensitive to change in pediatric health studies will be used to explore secondary and additional outcomes (see below). All online assessments will be delivered directly to participants, caregivers, and referring rheumatologists and collected via autonomous self-report in REDCap. The lead research coordinators will oversee data collection to ensure data is complete and collected in a timely manner.

### Primary outcomes

#### Recruitment/retention rates

Recruitment (the proportion of those approached to those who agree to participate; target >65%) and retention (the proportion of those who qualify to those who complete the study; target >80%) will be used as evidence to determine feasibility and acceptability of TEACH.

Qualitative data on TEACH feasibility/acceptability will also be gathered post-treatment by a trained graduate student of psychology. A semi-structured qualitative interview (~30 minutes) will be conducted with participants to assess feasibility and acceptability of the protocol.

### Secondary outcomes

#### Fatigue

Fatigue in the past seven days will be measured via the *PROMIS Pediatric Fatigue Short Form* or *Adult Short Form* [20, 21]. Adolescent participants will complete the pediatric short form which includes 10 items scored on a 5-point Likert scale (*0* = *never, 4* = *almost always*) to generate a score 0–40, with higher scores indicating more fatigue. Young adult participants will complete the adult short from that consists of eight items scored on a 5-point Likert scale (*1* = *not at all, 5* = *very much*) to create score 8–40, which are converted into T-scores. T-scores ≥ 60 are eligible.

#### Depressive symptoms

*The Children’s Depression Inventory 2nd Edition (CDI-II)* and *The Beck Depression Inventory-II (BDI-II)* assess depressive symptoms in the past two weeks for children and adults, respectively [22, 23]. The CDI-II consists of 28 items scored 0–2 for a total score 0–56. The BDI-II is a 21-item measure (scores 0–3) for a score 0–63, which can be transformed into a T-score. Higher scores on these measures indicate more severe symptoms. T-scores of ≥ 60 and ≤ 90 indicate eligibility.

#### Pain intensity

Average pain intensity over the past two weeks will be assessed using a *Visual Analog Scale (VAS),* a well-utilized measure of pain intensity [24, 25]. Values range from 0 (*no pain)* to 10 *(worst imaginable pain)* with a rating of ≥ 3 used [26] to indicate eligibility.

### Demographics

#### Demographic information

Age, sex, race, ethnicity, disease duration, family income, and caregiver occupation will be obtained. Caregivers will provide this information for adolescent participants. Information on the participant’s occupation, education, and residence will also be explored for those over age 18.

### Additional outcomes

#### Health-related outcomes

##### Disease activity & damage

Disease activity will be recorded by study rheumatologists via the *Systemic Lupus Erythematosus Disease Activity Index (SLEDAI-2 K)* [27] and the *Systemic Lupus International Collaborating Clinics Damage Index (SDI)* [28]. Scores on the SLEDAI-2 K range from 0 *(no activity)* to 105, with higher scores indicating greater disease activity, and scores on the SDI range 0–47, with higher scores indicating greater disease damage.

##### Medication use

Will be obtained via self/parent-report.

##### Medication adherence

Will be obtained through the *Medication Adherence Self-Report Inventory (MASRI)* [29], which consists of six items to measure missed medications.

##### Health-related quality of life

The *PedsQL Quality of Life Inventory *[30]* and PedsQL Rheumatology Module *[31] will assess participant quality of life. Participants under the age of 18 and their caregivers complete these measures. Items are scored on a 5-point Likert scale (*0* = *Never, 4* = *Almost Always).*

##### Pain interference

The *PROMIS Pain Interference Pediatric *[32, 33] *and Adult Form v1.0 Short Form *[34] will assess pain interference. The measure consists of eight questions on a 5-point Likert Scale ranging from 1 *(Not at all)* to 5 *(Very Much)*. A higher score represents higher pain inference in daily functioning.

### Mental health-related outcomes

#### Anxiety

The *Screen for Child Anxiety Related Disorders (SCARED)* is a self-report measure that assesses anxiety over the past three months [35]. Items include a 3-point Likert scale (*0* = *not true or hardly ever true, 2* = *very true or often true*) and totals range from 0–82. A score ≥ 25 indicates clinically significant anxiety.

#### Resilience

Will be measured using the *Brief Resilience Scale *[36]*,* a six item measure scored a 5-point Likert scale ranging from 1 = *Strongly disagree* to 5 = *Strongly Agree*. Items are averaged to create total possible scores 1–5, with higher scores indicating stronger resilience.

#### Adverse Childhood Experiences (ACEs)

*Adverse Childhood Experiences (ACEs)* is a nine-item form to assesses adverse events such as discrimination and violence experienced prior to age 18 [37]. It is completed directly by participants ages 18 years or older, or by caregivers for those under 18 years of age. Scores range 0-9, with higher scores indicating higher risk for adversities.

#### COVID-19-related distress

Participants will evaluate their average level of COVID-related distress *(0* = *no distress to 100* = *extreme distress)*, and (if appropriate) 2) the degree to which TEACH helped them cope with this distress *(0* = *not at all to 100* = *very much)*. COVID-related distress will be measured at each assessment point, whereas coping will only be administered after the TEACH program is completed. We created this measure for use in the study and it is comparable with how COVID-distress is measured using other approaches {Kazak, 2021 #174}.

#### Caregiver mental health

The *Depression Anxiety Stress Scales-21 (DASS-21) *[38] is a self-report measure that will assess caregiver mental health. The DASS-21 consists of 21 items that assess depression, stress, and anxiety. Items are rated from 0 *(Did not apply to me at all)* to 4 *(Applied to me very much, or most of the time),* with higher scores indicating more mental health symptoms.

### Daily diaries

Daily diaries will be completed by participants during the TEACH program to assess pain, mood, and fatigue. Each domain is assessed on a VAS, with qualitative end anchors (e.g., Pain = *No pain* to *Worst possible pain*; Mood = *Great* to *The worst;* Fatigue = *Not tired at all* to *Extremely tired)*.

### Adverse events

The Negative Effects Questionnaire, a 20-item questionnaire to examine adverse/unwanted events experienced during TEACH [39]. Participants will indicate whether they experienced the event and if it was related to TEACH. The degree of negativity is also measured on a 4-point Likert scale ranging from minimum to maximum.

### Intervention

#### The Treatment and Education Approach for Childhood-Onset Lupus (TEACH) Intervention

The TEACH intervention is a brief, remotely delivered intervention individualized to common cSLE symptoms and developed with patient/caregiver co-investigative feedback. It draws primarily from CBT strategies known to be effective in addressing fatigue [7], mood [8, 9], and pain symptoms [10, 11], and incorporates mindfulness meditation. Remotely delivered psychological therapy is considered to be as effective and more accessible to patients with co-morbid mood concerns than conventional in-person approaches [40].

TEACH was originally developed by the PI with her research team at Cincinnati Children’s Hospital Medical Center [18], and was modified from established CBT protocols for fatigue [41] and pain [42], with additional refinements made to address mood symptoms, developmental level (e.g., separate protocols for adolescents versus young adults), and other issues affecting those with cSLE (e.g., medication adherence). The research team partnered with five patients/caregiver co-investigators to further refine TEACH and create a telehealth adaptation.

For the current trial, TEACH will consist of six live one-hour HIPAA-compliant telehealth (Zoom) video sessions administered weekly (versus in person) with caregiver/partner support (see Table 1). During each session, an interventionist will teach new skills, assess home practice, answer questions, and help identify how participants will use the skills in their daily lives. Based on the refinement of TEACH, supplemental tools/practice activities will also be available to participants (via REDCap), and use of these tools will be tracked by the interventionist/research team. Licensed mental health providers and/or doctoral students under supervision in the US and Canada will serve as the interventionists. Every effort will be made to deliver TEACH consistently; however, in the case of technical difficulties or other access issues, interventionists will deliver TEACH using alternative strategies (e.g., phone call).

**Table 1:** TEACH protocol

Session	Attendee(s)	Adolescent Content	Young Adult Content
1	Participant, caregiver	Psychoeducation Caregiver guidelines	
2	Participant	Activity Pacing	
		Deep breathing Sleep hygiene	Pleasant activities Communication
3	Participant (and caregiver if < 18 years)	Muscle relaxation, Pleasant activities Communication	Relaxation strategies Medication adherence Sleep hygiene
4	Participant (and caregiver if ≥ 18 years)	Mindfulness Identifying automatic thoughts	
5	Participant	Challenging automatic thoughts Problem solving	
6	Participant, caregiver	Self-advocacy Maintenance	

For those < 18 years, caregiver refers to a parent or other legal guardian; for those 18 years and up, caregivers are optional and can include a parent, partner, or other significant support person. COVID distress and application of coping strategies to manage COVID-distress are mentioned throughout the protocol

At the start of the pandemic (March 2020), the co-investigative team met and unanimously decided to continue the study and modify the intervention to directly address COVID-19. Several TEACH interventionists (MRR, AD) and the PI (NRC) ( attended a training to implement safe telehealth through the American Psychological Association’s course, “Telepsychology Best Practice 101” [43], which was freely available in 2020 and provided education/training on ethical, legal, clinical, and technical issues/competencies to consider in telehealth practices. The team then developed their own study guidelines for telehealth based on the training. The TEACH protocol was also adjusted to include screening and assessment of the impact of COVID-19 on functioning (Table 1).

### Interventionist training

Interventionists will receive individual training from the PI (NRC) or other trained interventionists either in person or via Zoom conference. The protocol will be reviewed and practiced by interventionists, with direct feedback provided during training. Continued feedback/monitoring will be provided throughout. Interventionists will meet periodically via Zoom to discuss barriers and tips learned to ensure best practices.

#### Treatment fidelity

All sessions will be video-recorded and stored. Each primary site will have trained independent staff members who will complete an integrity rating for each session. The rating consists of a checklist to ensure consistent treatment content across all participants by the interventionist. Monthly interventionist meetings will be held to ensure consistency among treatment at US and Canada sites.

### Medical TAU

All participants across both conditions will continue standard medical treatment by their pediatric rheumatology team (TAU), either alone or in combination with TEACH.

### Data management and handling of missing data

Data will be managed and stored using REDCap, a secure software designed exclusively to support data capture for research studies. Considerable efforts (e.g., electronic data capture, standardized data checking strategies) will be made to limit missing data. Rates of missing data will be reported and addressed statistically if over 10%. If missing data are considered to be missing at random, multiple imputation or inverse probability weighting will be implemented at the time of final analysis. Given the modest sample size of this study, every effort will be made to minimize missing data.

### Statistical analysis plan

Recruitment/retention rates and qualitative analysis of the semi-structured interview will assess feasibility, tolerability, and acceptability of TEACH. Evidence of feasibility is demonstrated by > 65% of patients/families approached will agree to participate (H1) and > 80% of recruits will complete the TEACH protocol (H2). Participant feedback about TEACH will be solicited and will support program feasibility (H3). Recorded qualitative interviews will be transcribed for the team to identify and code themes and key content domains. Discrepancies between reviewers will be discussed at length until a consensus is reached. Members of the research team (NRC, AMK) are experts in qualitative data analysis [18, 44–48].

A repeated measures ANOVA will evaluate the hypotheses outlined in Aim 2. Participants will be allocated to the TEACH + TAU or TAU group. The mean change in fatigue (main secondary outcome), depressive symptoms, and pain from baseline to eight-week post will be analyzed separately to determine the effect of TEACH on these symptoms in comparison to the control group.

#### Exploratory Outcomes 

Given that data from exploratory variables will be collected throughout the study, additional investigations into the effect of TEACH on these variables will be conducted. Long-term outcomes measured at the 20-week assessment of TEACH will be evaluated.

#### Sample Size Estimate

The sample size estimate for this was based on a two-sided test for the averaged difference between two group means (for fatigue) using a repeated measures ANOVA design with a significance level of *p* < 0.05. A minimum sample size of 60 participants (30 per treatment group) and an effect size of 0.6 [18] results in 76% power. Given that an estimated 80% of participants will be retained in the study, a total of 75 participants will need enroll in the study.

## Discussion

cSLE is a chronic, debilitating autoimmune disease associated with more severe symptoms as compared to adult-onset SLE and a 20-fold increased risk of mortality [49]. cSLE is commonly associated with fatigue, mood issues, and pain, which contribute to poor health-related quality of life [2, 3]. Given these common symptoms are modifiable with nonpharmacological therapies, CBT may optimize patient outcomes as part of a multi-disciplinary approach to care [50]; however, there is mixed evidence to date that CBT manages mood and related symptoms in children [18, 51] and adults [13] with SLE. A tailored intervention such as TEACH using evidence-based cognitive behavioral and mindfulness meditation techniques to manage fatigue, depressive symptoms and pain may be needed to address concerns specific to this population.

While we have demonstrated feasibility of TEACH in a pilot investigation [18], a rigorous and well-controlled trial of this promising approach is critically needed to advance understanding and improve outcomes for youth with cSLE. TEACH is a brief tailored therapy designed to improve fatigue, mood, and pain in youth with cSLE. It is important to assess if TEACH is feasible and associated with improvements in these symptoms following treatment as compared to standard medical care. Once these effects are established, we will investigate additional factors such as patterns/predictors of treatment response.

COVID-19 impacted the study planning and recruitment plan. As such, COVID-related distress will now be measured and targeted. Furthermore, we expect enrollment rates to vary during different periods of the COVID-19 pandemic. We will examine these data carefully in the context of when it was collected.

We anticipate minimal risk and maximal benefits to patients/families. The present trial allows for understanding of treatment effects. Lack of active treatment comparator and a modest sample size are limitations. In the future, we plan to test the *implementation* of TEACH in a larger scale multi-site investigation and assess sustainability of treatment effects. Assuming TEACH is effective and future implementation efforts are successful, we will share the program with rheumatology providers to integrate TEACH into routine care. Future work will also examine mechanisms of treatment response and whether modifications (e.g., booster sessions) are needed.

Since cSLE is a complex condition disproportionately impacting females of color, it is important to test a treatment tailored to meet the needs of these individuals. Given the current trial focuses on cSLE, the results will be most applicable to this population. However, the learnings of this trial may provide insights into to developing and testing treatments for other conditions.

This RCT was funded by the CARRA-AF organization, a network consisting of rheumatologists, other professionals, patients, and their caregivers who all take an interest in research. Aligned with the mission of CARRA, the investigative team partnered with patients with lupus and their caregivers to amplify their voice in the refinement of TEACH. In addition, several of the study investigators (NRC, TR, AMK) co-chair the mental health workgroup of CARRA. This is noteworthy as our CARRA mental health workgroup is finalizing guidance statements to assess and manage mental health concerns in pediatric rheumatology care. Given the synchrony of our CARRA grant-funded research and the larger body of work within the CARRA mental health workgroup, we anticipate continuing to leverage the organization and its partners for future research.

## Conclusion

In conclusion, this is the first RCT of a remotely delivered, tailored psychological treatment to target fatigue, mood symptoms, and pain in youth with cSLE. If successful, TEACH has the potential to positively impact the standard of care of youth with cSLE.

## Data Availability

Not applicable.

## Abbreviations

ACEs: Adverse Childhood Experiences
BDI-II: Beck Depression Inventory-II
CAN: Canada
CARRA: Childhood Arthritis and Rheumatology Research Alliance
CARRA-AF: Childhood Arthritis and Rheumatology Research Alliance – Arthritis Foundation
CBT: Cognitive Behavioral Therapy
CDI-II: Children’s Depression Inventory 2nd Edition
cSLE: Childhood-onset Systemic Lupus Erythematosus
DASS-21: Depression Anxiety Stress Scales-21
MASRI: Medication Adherence Self-Report Inventory
MSU: Michigan State University
RCT: Randomized controlled trial
SCARED: Screen for Child Anxiety Related Disorders
SDI: Systemic Lupus International Collaborating Clinics Damage Index
SLEDAI-2K: Systemic Lupus Erythematosus Disease Activity Index 2000
TAU: Treatment as usual
TEACH: Treatment and Education Approach for CHildhood-onset Lupus
US: United States
VAS: Visual Analog Scale
